# Bone Quality and Fractures in Women With Osteoporosis Treated With Bisphosphonates for 1 to 14 Years

**DOI:** 10.1002/jbm4.10549

**Published:** 2021-09-21

**Authors:** Hartmut H Malluche, Jin Chen, Florence Lima, Lucas J Liu, Marie‐Claude Monier‐Faugere, David Pienkowski

**Affiliations:** ^1^ Division of Nephrology, Bone & Mineral Metabolism, Department of Internal Medicine University of Kentucky Lexington KY USA; ^2^ Division of Biomedical Informatics, Department of Internal Medicine University of Kentucky Lexington KY USA; ^3^ Department of Computer Science University of Kentucky Lexington KY USA; ^4^ F. Joseph Halcomb III, MD Department of Biomedical Engineering University of Kentucky Lexington KY USA

**Keywords:** BONE QUALITY, OSTEOPOROSIS, BISPHOSPHONATES, DXA, FRACTURE RISK ASSESSMENT

## Abstract

Oral bisphosphonates are the primary medication for osteoporosis, but concerns exist regarding potential bone‐quality changes or low‐energy fractures. This cross‐sectional study used artificial intelligence methods to analyze relationships among bisphosphonate treatment duration, a wide variety of bone‐quality parameters, and low‐energy fractures. Fourier transform infrared spectroscopy and histomorphometry quantified bone‐quality parameters in 67 osteoporotic women treated with oral bisphosphonates for 1 to 14 years. Artificial intelligence methods established two models relating bisphosphonate treatment duration to bone‐quality changes and to low‐energy clinical fractures. The model relating bisphosphonate treatment duration to bone quality demonstrated optimal performance when treatment durations of 1 to 8 years were separated from treatment durations of 9 to 14 years. This may be due to a change in relationship of bone‐quality parameters with treatment duration. This model also showed that the effects of bisphosphonate treatment duration were most highly correlated with changes in means and standard deviations of infrared spectroscopically derived mineral and matrix parameters and histomorphometric bone turnover parameters. A second model related treatment duration to bone fracture in all 22 patients who fractured while on treatment with bisphosphonates for more than 8 years. This second model showed that bisphosphonate treatment duration, not hip bone mineral density (BMD), was the most strongly correlated parameter to these low‐energy bone fractures. Application of artificial intelligence enabled analysis of large quantities of structural, cellular, mineral, and matrix bone‐quality parameters to determine relationships with long‐term oral bisphosphonate treatment and fracture. Infrared spectroscopy provides clinically relevant bone‐quality information of which bone mineral purity is among the most relevant. Nine or more years of bisphosphonate treatment was associated with abnormal bone mineral purity, matrix abnormalities, and low‐energy fractures. These data justify limiting bisphosphonate treatment duration to 8 years. © 2021 The Authors. *JBMR Plus* published by Wiley Periodicals LLC on behalf of American Society for Bone and Mineral Research.

## 1 Introduction

Osteoporosis is a health problem of major proportions. It affects more than 200 million people worldwide, results in more than 2 million fractures annually in the US, and hospital admissions for osteoporotic fractures exceed those of heart attacks, strokes, and breast cancer combined.^(^ ^1^, ^2^, ^3^ ^)^ Osteoporotic fractures may occur because of loss of bone quantity and—less widely recognized—unfavorable changes to bone quality.^(^ ^4^, ^5^ ^)^ Bisphosphonates are the primary modality for treating postmenopausal osteoporosis; their effectiveness is due to reduction of osteoclastic activity accompanying high bone turnover.^(^ ^6^ ^)^ Concerns exist that bisphosphonate use may be associated with bone‐quality changes that reduce load‐bearing mechanical competence and this may partially explain reported occurrences of low‐energy fractures accompanying long‐duration bisphosphonate use.^(^ ^7^ ^)^ These reports dampen the enthusiasm of patients and physicians for use of bisphosphonates to treat osteoporosis.

Evidence supporting the hypothesis of a relationship between long‐term bisphosphonate use and bone‐quality changes was provided in our prior studies of bone structure^(^ ^8^ ^)^ and mechanical properties.^(^ ^9^ ^)^ These structural and material property changes may be the manifestation of bisphosphonate‐related changes in bone cell activity and bone mineral or matrix structural or compositional changes. Support for this hypothesis is found in other studies suggesting changes in relative mineralization, crystallinity, and crystal size or collagen cross‐linking with bisphosphonate use.^(^ ^10^, ^11^ ^)^ This is shown through changes in the distribution, but not mean values, of these parameters with varying bisphosphonate treatment duration. This may be due to method of analysis, small numbers of study patients, limited treatment duration, or small changes in bone‐quality parameters with bisphosphonate treatment duration.^(^ ^12^ ^)^ Furthermore, the relationship between bisphosphonate treatment duration and low‐energy bone fractures is also unclear. Thus, the objectives of this study were to: (i) evaluate structural, cellular, turnover, mineral and matrix parameters of bone, and relevant patient data and their relationship with 1 to 14 years of bisphosphonate treatment duration and (ii) to determine relationships between bone fracture and structural, cellular, turnover, mineral and matrix parameters of bone, relevant patient data, and bisphosphonate treatment duration.

## 2 Materials and Methods

### 2.1 Study design

Relationships among bone mineral, matrix, structural, and dynamic properties with continuous oral bisphosphonate treatment duration were evaluated from human anterior iliac crest bone samples using histomorphometry and Fourier transform infrared spectroscopy (FTIR) with a cross‐sectional design. FTIR is an established technique for analyzing various tissues in health and disease; it is especially useful for quantifying various bone mineral and matrix parameters reflecting bone quality and fracture resistance.^(^ ^13^, ^14^, ^15^, ^16^ ^)^ Bone mineral parameters included mean values and standard deviations of the mineral to matrix ratio, carbonate to phosphate ratio, and c‐axis mineral crystal length (crystallinity). The bone matrix parameter was the ratio of mature to immature collagen cross‐links (cross‐linking ratio). Histomorphometric‐based bone structure parameters were bone volume/tissue volume, trabecular separation, and trabecular thickness. Histomorphometric‐based bone formation parameters were osteoid volume/bone volume, osteoid surface/bone surface, osteoid thickness, number of osteoblasts/bone perimeter, erosion surface/bone surface, number of osteoclasts/bone perimeter, mineral apposition rate, mineralizing surface/bone surface, bone formation rate/bone surface, mineralization lag time, and activation frequency. Clinical and biochemical data were obtained from the patient's medical records available at time of biopsy and were also stored in the Kentucky Bone Registry. Design of this study conformed to the Declaration of Helsinki and was approved by the University of Kentucky Institutional Review Board.

### 2.2 Patients

This was a retrospective cross‐sectional study of bone samples selected from among a population collected from the past three decades and stored in the registry. The included bone samples were from a consecutive series of adult female patients with osteoporosis previously treated with oral bisphosphonates (alendronate, risedronate, or ibandronate) who presented to the University of Kentucky Metabolic Bone Disease Clinic for turnover evaluation to decide optimal therapy continuation. These patients were previously diagnosed by dual‐energy X‐ray absorptiometry (DXA) of the femoral neck or lumbar spine or by low‐energy fracture regardless of DXA score and were offered a minimally invasive anterior iliac crest bone biopsy with double tetracycline labeling for workup of turnover for choice of therapy. Bone biopsies are routinely offered by our clinic to all patients with metabolic bone disorders; approximately 85% agree to the biopsy. All study patients signed an Institutional Review Board–approved consent form permitting their results to be used for subsequent scientific studies. Low‐energy fractures were defined as radiologically observed fractures occurring in the absence of documented trauma.

### 2.3 Inclusion and exclusion criteria

The inclusion criteria were adult (age older than 21 years) female patients with osteoporosis who were treated with oral bisphosphonates for at least 1 year and had verified intake of double‐tetracycline labeling. The exclusion criteria were genetic diseases (such as osteogenesis imperfecta, hypophosphatemic rickets, etc.), chronic kidney or liver diseases, primary hyperparathyroidism, neoplasms, or previous treatment with medications known to alter bone metabolism (except oral bisphosphonates). Patients presently or previously treated with anticonvulsants or long‐term steroids or those with documented chronic alcoholism, drug addiction, malabsorption, malignancy, bariatric surgery, Paget's disease, osteogenesis imperfecta, hemiplegia or paraplegia, organic illness with potential influence on bone metabolism, or uncontrolled systemic illness were also excluded.

### 2.4 Bone biopsy, histology, and histomorphometry

Before bone biopsy, patients received oral demeclocycline hydrochloride (300 mg) twice daily for 2 days, followed by a 10‐day tetracycline‐free interval and another course of tetracycline hydrochloride (250 mg) twice daily for 4 days. Anterior iliac crest bone biopsies were performed under local anesthesia after an additional 4 days. Iliac crest bone samples were fixed with ethanol at room temperature, dehydrated, and embedded in polymethylmethacrylate as described previously.^(^ ^17^ ^)^ Serial sections of 3‐μm and 7‐μm thickness were cut with a microtome equipped with a carbide‐edged knife. Sections were stained with modified Masson‐Goldner trichrome stain. Unstained sections were prepared for phase‐contrast and fluorescence light microscopy. Bone histomorphometry for static and dynamic parameters of bone structure, formation, and resorption was performed at a magnification of 200× using the Osteoplan II system (Carl Zeiss, New York, NY, USA). All measured histomorphometric parameters complied with the recommendations of the nomenclature committee of the American Society for Bone and Mineral Research.^(^ ^18^, ^19^ ^)^

### 2.5 Infrared spectroscopy

Two to five 4‐μm‐thick sections were cut from each embedded bone sample, and each section was individually sandwiched between two barium fluoride discs. The sandwiched sample was placed on the stage of a Nexus 6700 Fourier Transform Infrared Spectrometer (Thermo Electron, Waltham, MA, USA). Infrared spectra were collected over most of the trabecular bone area of each sample using an automated routine. Separate background scans were done to enable spectral subtraction and correction of errors attributable to the environment, BaF discs, and embedding material.^(^ ^20^ ^)^

### 2.6 Fourier transform infrared spectroscopic image analysis

**Fig 1:**
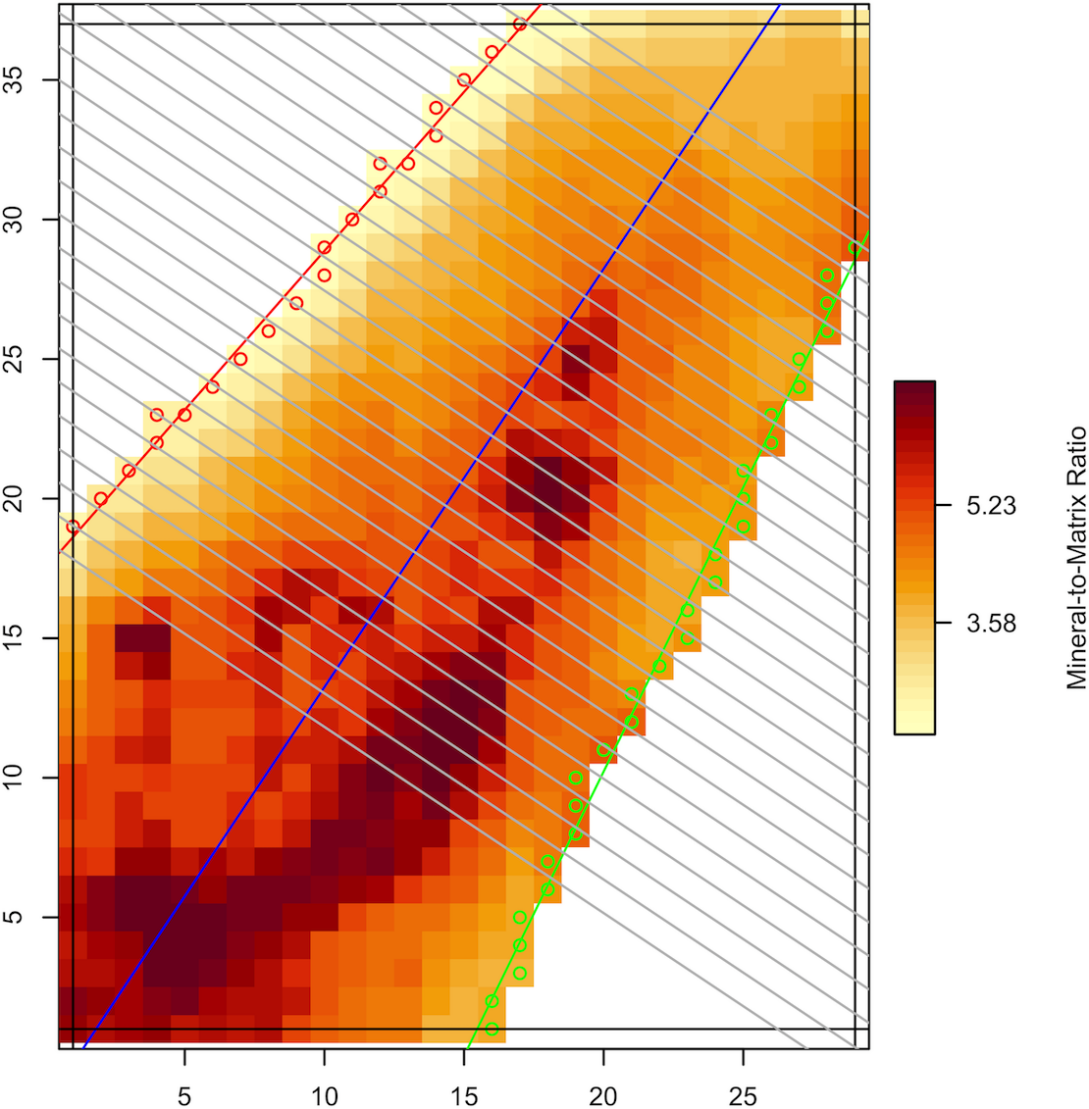
Mineral to matrix ratio on trabecular bone surface. Representative surface of a single trabeculum and grid guiding systematic Fourier transform infrared spectroscopic (FTIR) measurements. Variations in the mineral to matrix ratio (the bone mineral quality parameter shown) are denoted using different color shades. Dark red denotes bone surface with high mineral to matrix ratio; light orange denotes bone surface with low mineral to matrix ratio. The thin dark line defining the longitudinal axis of the trabeculum references subsequently constructed multiple orthogonal lines along which FTIR scans were made each 6 μm. Red and green circles denote trabecular boundaries. Background area (white region) was excluded. All mineral and matrix properties were measured along the orthogonal lines with a 36‐μm square area resolution.

Analysis of these spectroscopic images began by automated identification of the area of bone scanned by the infrared beam. This area was defined as the sum of 6 μm × 6 μm scanned pixels. Location of these pixels were determined from a grid established by passing a straight line along the longitudinal axis of the trabeculum and then arranging a series of parallel lines spaced 6 μm apart along this line. These parallel lines ranged in length from one edge of a trabeculum to the opposite edge of the same trabeculum within the field of view (Fig. 1). Values for the mineral and matrix response parameters, ie, mineral to matrix ratio, carbonate to phosphate ratio, c‐axis mineral length, and cross‐linking ratio, were calculated from the scans taken in the area defined by these lines (Fig. 1). These values were determined for each 6 μm × 6 μm area on the surface of each examined trabeculum using reported techniques.^(^ ^20^ ^)^ Mean values and standard deviations of each spectroscopic parameter within each sample were determined. Approximately 325 individual bone areas, each 6 μm × 6 μm, were scanned per patient.

Measured mineral and matrix parameters were divided into low, medium, or high categories. Thresholds for these categories were as follows: Low were those values less than the mean value of the lower half minus 1 SD, high were those greater than the mean value of the greater half plus 1 SD, and the medium category consisted of all measurements above low but below high. Specific values denoting the low or high thresholds are shown for each of the mineral and matrix parameters (Table 1).

**Table 1:** Threshold Values for Categorization of FTIR Parameters

FTIR parameter	Low category threshold	High category threshold
Mineral to matrix ratio	3.580	5.230
Carbonate to phosphate ratio	0.008	0.010
C‐axis mineral crystal length	1.072	1.164
Cross‐linking ratio	3.065	3.769

FTIR = Fourier transform infrared spectroscopy.

The total area of trabecular bone scanned by the infrared beam was calculated for each sample. The mean value of each parameter in each category (Table 1) was normalized by the total bone area from which that value was obtained. Mean and standard deviations of these area‐normalized values were then determined. The analyses extracted a total of 44 infrared spectroscopy‐based parameters and 12 histomorphometric parameters. These parameters were analyzed using statistical methods and machine‐learning modeling to quantify the relationship between bone quality and bisphosphonate treatment duration.

### 2.7 Statistics and machine‐learning modeling

The goal of machine‐learning techniques was to generate the best prediction of an independent variable from a variety of dependent variables. Once a satisfactory prediction was obtained, machine‐learning methods were then used to identify the parameters that contributed most to model performance. Statistical and machine‐learning analyses used these infrared spectroscopy, histomorphometry, and patient‐based parameters to understand relationships among bone quality, bisphosphonate treatment duration, and fracture. Two computational challenges were addressed. The first challenge arose from the disparity between the large number of parameters analyzed compared with the number of study subjects. This disparity frequently leads to the computationally difficult^(^ ^21^ ^)^ problem of data overfitting that besets construction of robust machine‐learning models. To overcome this challenge, XGBoost, an established and optimized distributed gradient boosting algorithm,^(^ ^22^ ^)^ was adapted from the library of R statistical software.^(^ ^23^ ^)^ By implementing conventional machine‐learning algorithms under the Gradient Boosting framework,^(^ ^24^ ^)^ XGBoost offered superior regularized model formalization to control overfitting and was thus capable of providing parallel‐tree boosting that effectively solves this challenge. Before employing XGBoost, a minimum redundancy/maximum relevance feature reduction method (mRMR) was used to identify the most critical features in each candidate model based upon the data.^(^ ^25^ ^)^

**Fig 2:**
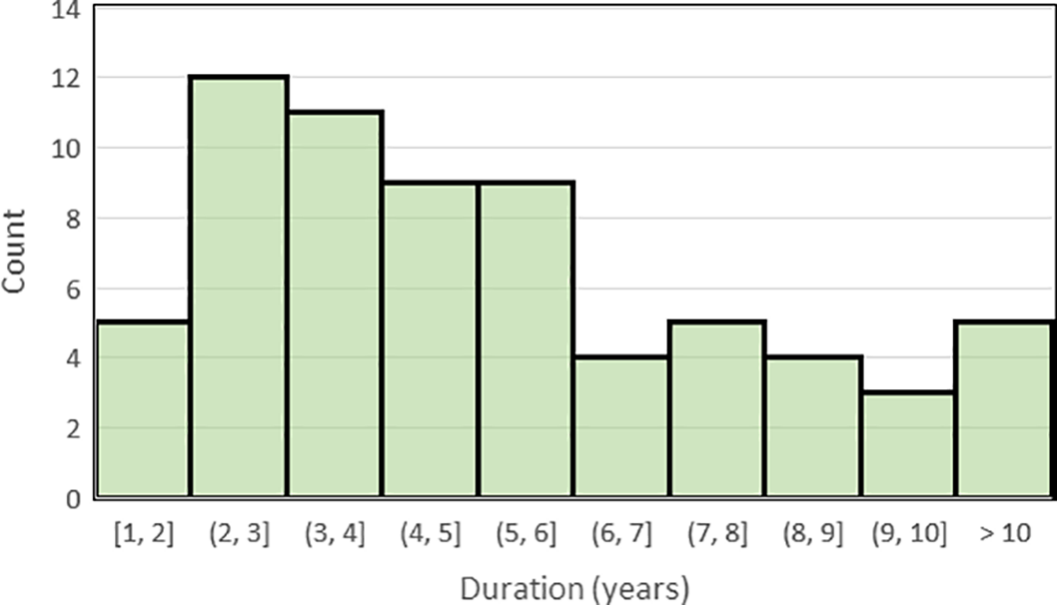
Distribution of bisphosphonate treatment durations among the study subjects.

The second challenge arose from the lack of a uniform or normally distributed bisphosphonate treatment duration (Fig. 2). Clearly, more patients were treated for short durations compared with those treated for long durations. Although a slight imbalance is generally of no concern, challenges arise in machine‐learning if imbalances in the data are substantial. This challenge was resolved using repetitive random down‐sampling^(^ ^26^ ^)^ to generate a balanced set (equal numbers of patients in each bisphosphonate treatment duration category) of training data from the original data set. Cross‐validation was used to reduce random effect. Data analysis was performed using R version 3.6.3 (R Foundation for Statistical Computing) and the R package XGBoost. Model parameters were tuned using the caret package in R.

To support the applicability of the sample size used and the generalizability of the models developed, the effort focused on bone‐quality parameters with minimum redundancy and maximum relevance. In addition, the XGBoost model was made more conservative by restricting the maximum depth of trees, tuning the XGBoost parameters γ and η to minimize loss reduction and step size shrinkage, as well as utilizing L1 and L2 regularization techniques.

## 3 Results

### 3.1 Subjects

Pertinent characteristics of the 67 included study patients are shown (Table 2). Analysis of the relationships between bone‐quality changes and bisphosphonate treatment duration was non‐trivial due to the plethora of data obtained. For this reason, a meta‐analysis machine‐learning model was developed using a two‐step process. The first step involved consideration of the range of observed bisphosphonate treatment durations as a two‐category discrete variable in which the categories were separated by varying treatment durations. The separation point distinguishing these two categories varied from 5 to 9 years. Fit of this model, assessed using the area under the curve (AUC) of the receiver operating characteristic (ROC) metric, was optimized (AUC = 0.88) when the bisphosphonate treatment duration categories were distinguished at the 8‐year time point (Table 3). That is, the relationship between changes in bone‐quality parameters and bisphosphonate treatment duration was optimal when treatment duration was separated into two categories, ie, from 1.1 years to the end of 8 years and 9 years to the maximum of 14 years.

**Table 2:** Pertinent Characteristics of the 67 Study Subjects at Time of Iliac Crest Bone Biopsy

	Mean	Standard deviation	Median	Minimum	Maximum
Age (years)	60	8	59.00	34	77
BMD spine (*t* value)	−2.48	0.97	−2.50	−4.20	0.70
BMD hip (*t* value)	−1.90	0.88	−2.05	−4.10	1.00
Body mass index	25.08	5.13	23.51	15.96	43.53
Duration of bisphosphonate treatment (years)	5.60	2.95	5.00	1	14

BMD = bone mineral density.

**Table 3:** Model Performance Resulting From a Meta‐Analysis Relating Bone Parameters to Bisphosphonate Treatment Duration

Bisphosphonate treatment duration categories (years)	Model fit parameters		First treatment period			Second treatment period		
	AUC	Accuracy	Precision	Sensitivity	F‐1	Precision	Sensitivity	F‐1
1–5 and 6–14	0.71	0.71	0.77	0.59	0.67	0.67	0.82	0.74
1–6 and 7–14	0.75	0.73	0.70	0.79	0.74	0.77	0.67	0.71
1–7 and 8–14	0.77	0.76	0.92	0.59	0.71	0.69	0.94	0.80
**1–8 and 9–14**	**0.88**	**0.87**	**0.93**	**0.82**	**0.86**	**0.84**	**0.92**	**0.87**
1–9 and 10–14	0.82	0.89	0.88	0.90	0.89	0.91	0.88	0.89

AUC = area under the receiver operating characteristic curve.

F‐1 denotes the harmonic mean of precision and sensitivity. Bone parameters used in the model consist of infrared spectroscopic parameters, histomorphometric parameters, and body mass index. Boldface denotes treatment duration categories with optimal model performance.

The second step of this process involved use of the identified optimal two duration categories, 1 to 8 and 9 to 14 years of bisphosphonate treatment. The bone‐quality machine‐learning model was then optimized by sequential inclusion of various infrared spectroscopic and histologic bone‐quality parameters as well as various patient parameters (Table 4). Differentiation between the resulting models was then accomplished using the AUC metric. The results showed that the best performance (AUC = 0.90) was obtained using three different types of parameters: Two were infrared spectroscopic and histomorphometric and one was a patient‐specific parameter (bone mineral density [BMD] hip). The results of these sequential analyses are shown in ascending order (Table 4).

**Table 4:** Results of Candidate Machine‐Learning Models Relating Categorical Bisphosphonate Treatment Duration to the Various Parameter Types Shown

Parameter type	Model fit parameters		First treatment period			Second treatment period		
	AUC	Accuracy	Precision	Sensitivity	F‐1	Precision	Sensitivity	F‐1
Histomorphometry	0.60	0.63	0.67	0.56	0.61	0.60	0.70	0.65
Infrared spectroscopy	0.70	0.72	0.82	0.56	0.66	0.67	0.88	0.76
Infrared spectroscopy and histomorphometry	0.86	0.85	0.96	0.74	0.83	0.80	0.96	0.87
Infrared spectroscopy, histomorphometry, and patient age	0.88	0.84	0.91	0.76	0.82	0.80	0.92	0.85
Infrared spectroscopy, histomorphometry, and patient BMI	0.88	0.87	0.93	0.82	0.86	0.84	0.92	0.87
Infrared spectroscopy, histomorphometry, and patient BMD hip and BMI	0.89	0.87	0.84	0.92	0.87	0.93	0.82	0.87
**Infrared spectroscopy, histomorphometry, and BMD hip**	**0.90**	**0.88**	**0.83**	**0.96**	**0.89**	**0.96**	**0.80**	**0.87**

AUC = area under the receiver operating characteristic curve; BMI = body mass index; BMD = bone mineral density.

F‐1 denotes the harmonic mean of precision and sensitivity. Boldface denotes the parameters associated with optimal machine‐learning performance of the duration model.

### 3.2 Machine‐learning duration model

**Fig 3:**
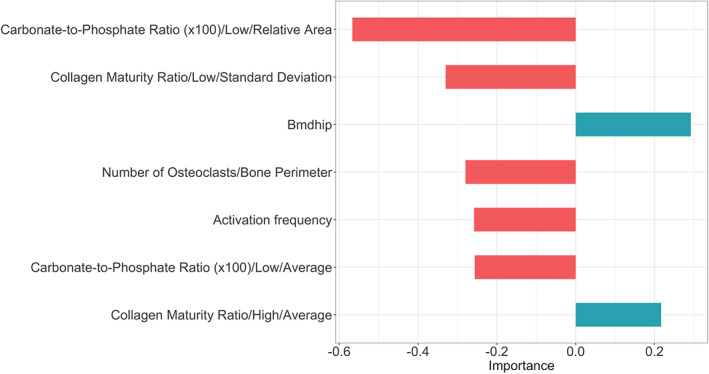
Relative importance and relationship of various bone relevant parameters to bisphosphonate treatment duration as determined by the machine‐learning model. This model is referred to in the text as the “duration model.” Length of the horizontal bars denotes the magnitude of the linear coefficient in the machine‐learning duration model; direction and color of the bars denote the sign of the coefficient. Red bars extending to the left denote coefficients negatively correlated with increasing bisphosphonate treatment duration; teal bars extending to the right denote coefficients positively correlated with increasing bisphosphonate treatment duration.

The relationship between bisphosphonate treatment duration and all infrared spectroscopic, histomorphometric, and patient‐related parameters were analyzed using machine‐learning (Supplemental Fig. S1). Only those parameters with estimated linear coefficients exceeding 0.2 were retained in a model linking bisphosphonate treatment duration with bone quality. The retained parameters include parameters of mineral perfection, collagen maturity, histologic cell and turnover parameters, and BMD of the hip (Fig. 3).

**Fig 4:**
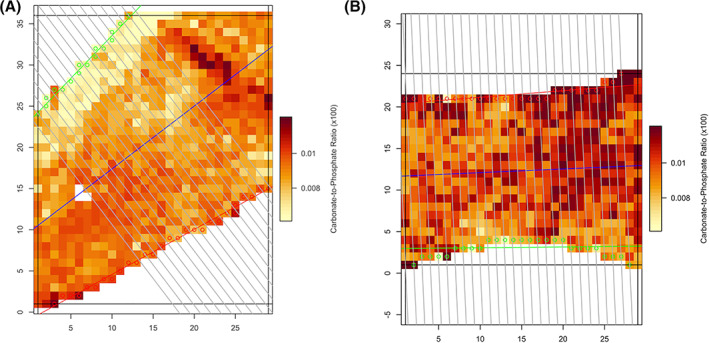
(*A*) Images from FTIR examination of prepared trabecular bone surfaces. These images colorimetrically depict varying carbonate to phosphate ratios in individual 6 μm × 6 μm square areas of trabecular bone surface. Greater color intensity denotes bone surfaces with greater values of the carbonate to phosphate ratio. These images were obtained from different study patients each aged 59 years. The left image was obtained from a patient treated with bisphosphonates for 3 years, the right image from a patient treated for 10 years. The image from the longer bisphosphonate treatment duration shows more pixels with greater carbonate to phosphate ratio. (*B*) Images from FTIR examination of prepared trabecular bone surfaces. These images colorimetrically depict varying carbonate to phosphate ratios in individual 6 μm × 6 μm square areas of trabecular bone surface. Greater color intensity denotes bone surfaces with greater values of the carbonate to phosphate ratio. These images were obtained from different study patients each aged 59 years. The left image was obtained from a patient treated with bisphosphonates for 3 years, the right image from a patient treated for 10 years. The image from the longer bisphosphonate treatment duration shows more pixels with greater carbonate to phosphate ratio.

**Fig 5:**
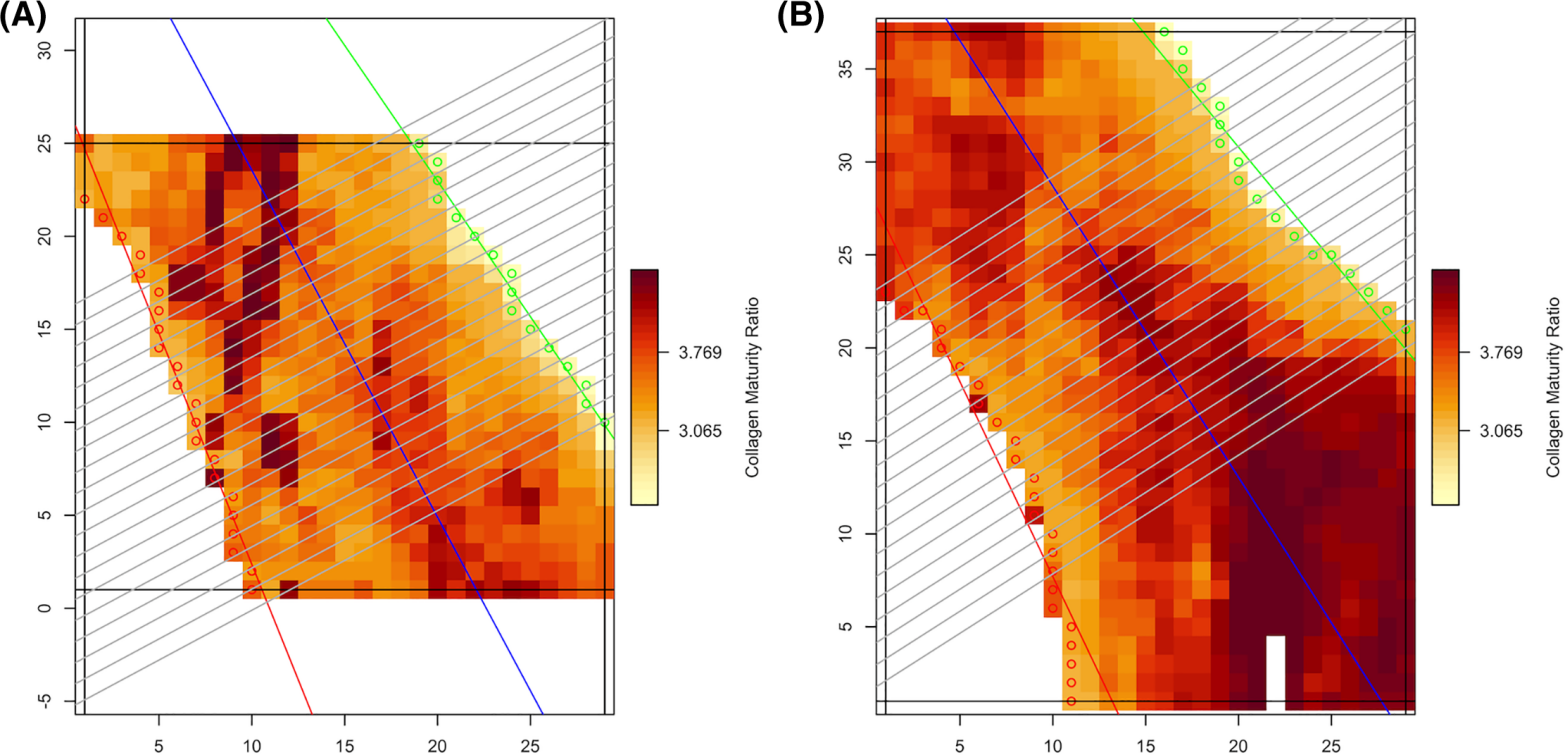
(*A*) Images from FTIR examination of prepared trabecular bone surfaces depicting ratios of mature to immature collagen cross‐links. Darker colors represent bone surfaces with greater ratios of mature to immature collagen cross‐links. Both images were obtained from study patients aged 63 years. The left image was obtained from a patient treated with bisphosphonates for 4 years, the right image from a patient treated for 12 years. The image from the patient treated for 12 years shows an increased ratio of mature to immature collagen cross‐links. (*B*) Images from FTIR examination of prepared trabecular bone surfaces depicting ratios of mature to immature collagen cross‐links. Darker colors represent bone surfaces with greater ratios of mature to immature collagen cross‐links. Both images were obtained from study patients aged 63 years. The left image was obtained from a patient treated with bisphosphonates for 4 years, the right image from a patient treated for 12 years. The image from the patient treated for 12 years shows an increased ratio of mature to immature collagen crosslinks.

Two of the parameters shown in Fig. 3 express changes in mean values of bone mineral parameters and two parameters express changes in mean values or standard deviations of bone matrix parameters. Examples of actual infrared spectroscopic images from which bone mineral and matrix quality data were obtained, ie, the carbonate to phosphate ratio and matrix maturity (ratio of mature to immature collagen cross‐links) are shown (Figs. 4*A*, *B* and 5*A*, *B*).

### 3.3 Machine‐learning fracture model

Twenty‐two^(^ ^22^ ^)^ study patients sustained nontraumatic fractures during bisphosphonate treatment (Tables 5 and 6). Machine‐learning methods were again employed to analyze the relationship between all studied bone parameters and bisphosphonate treatment in these 22 patients. The presence or absence of bone fracture was modeled as a function of both categorical and continuous bisphosphonate treatment duration, and bone quality measured using infrared spectroscopic, histomorphometric, and patient‐related parameters. The AUC metric was again used to determine the parameters that provide the best fit with fracture (Table 7). Optimum model performance (AUC = 0.88) for overall fit to the presence or absence of bone fractures was obtained when bisphosphonate treatment duration (continuous variable) and bone‐quality parameters (infrared spectroscopic and histomorphometric) were included in the model (Table 7). This model was dominated by bisphosphonate treatment duration (continuous) and the infrared spectroscopic bone‐quality parameters; inclusion of the histomorphometric parameters contributed little to the model.

**Table 5:** Pertinent Characteristics of Patients Who Fractured While on Bisphosphonate Treatment (*n* = 22)

	Mean	Standard deviation	Median	Minimum	Maximum
Age at time of biopsy (years)	61	9	61.50	34.00	77.00
BMD hip (*t* value)	−1.90	0.77	−1.90	−3.50	−0.50
Body mass index	25.57	4.83	25.49	18.75	33.80
Duration of treatment (years)	7.28	3.36	6.50	2.50	14.00

BMD = bone mineral density.

**Table 6:** Pertinent Characteristics of Patients Who Did Not Fracture While on Bisphosphonate Treatment (*n* = 45)

	Mean	Standard deviation	Median	Minimum	Maximum
Age at time of biopsy (years)	59	8	59	39	77
BMD hip (*t* value)	−1.90	0.93	−2.10	−4.10	1.00
Body mass index	25.06	5.43	23.74	15.96	43.53
Duration of treatment (years)	4.81	2.35	4.00	1.00	11.33

BMD = bone mineral density.

**Table 7:** Results of Candidate Machine‐Learning Models Relating Bone Fractures in Patients Receiving Bisphosphonate Treatment to Various Parameter Types

Parameter type	Model fit parameters		No fracture			Fracture		
	AUC	Accuracy	Precision	Sensitivity	F‐1	Precision	Sensitivity	F‐1
Histomorphometry	0.44	0.55	0.67	0.58	0.55	0.47	0.50	0.49
Duration (categorical)	0.60	0.84	0.81	1.00	0.89	1.00	0.60	0.71
Duration (continuous)	0.72	0.71	0.77	0.73	0.75	0.62	0.67	0.64
Infrared spectroscopy	0.78	0.74	0.90	0.65	0.76	0.62	0.89	0.73
Infrared spectroscopy and histomorphometry	0.86	0.81	0.88	0.79	0.84	0.73	0.83	0.78
Infrared spectroscopy and duration (continuous)	0.86	0.78	0.86	0.77	0.81	0.70	0.80	0.74
**Infrared spectroscopy and histomorphometry and duration (continuous)**	**0.90**	**0.83**	**0.96**	**0.76**	**0.84**	**0.72**	**0.94**	**0.81**

AUC = area under the receiver operating characteristic curve.

F‐1 denotes the harmonic mean of precision and sensitivity. Boldface denotes the parameters associated with optimal machine‐learning performance of the fracture model.

**Fig 6:**
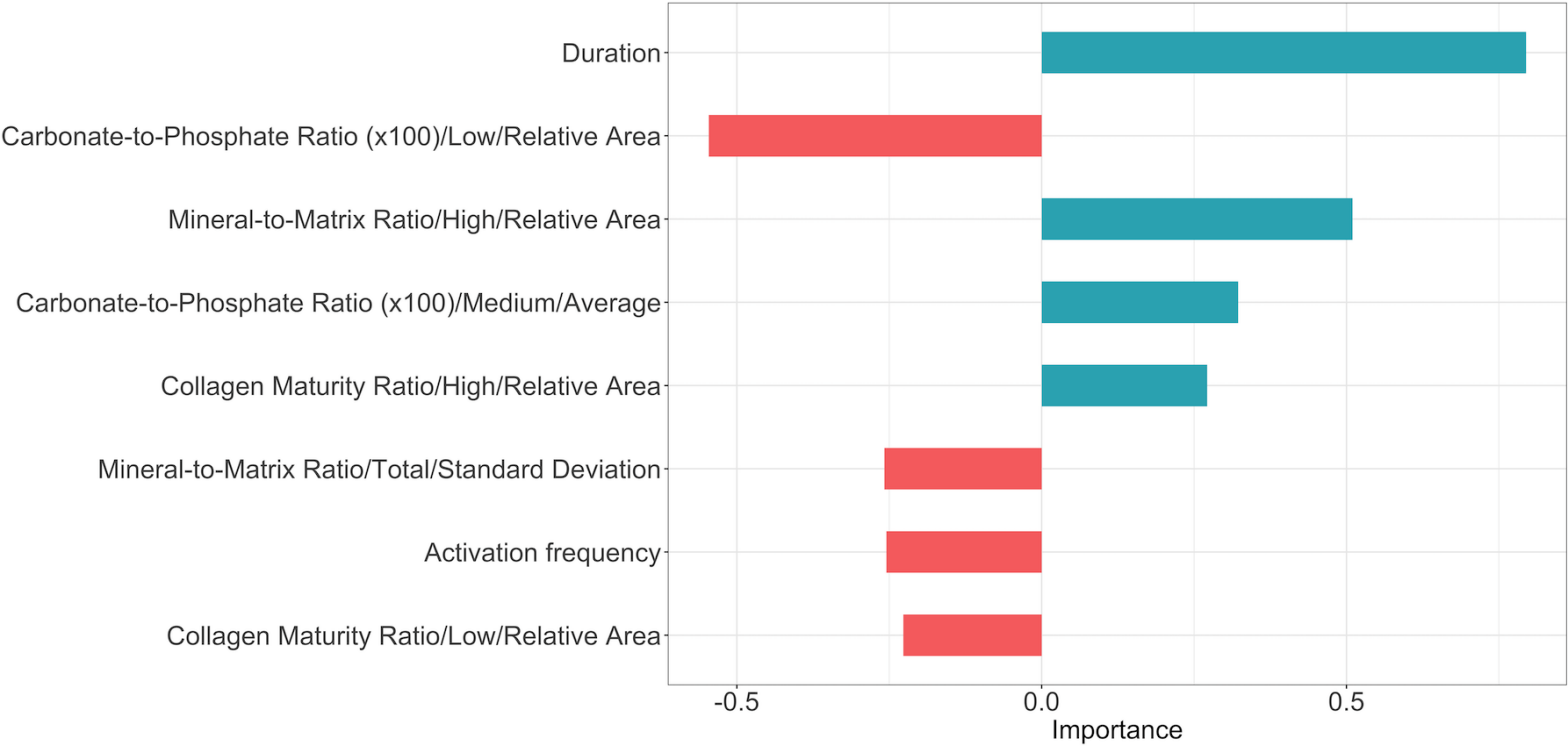
Relative importance and relationship of various bone parameters to fracture in bisphosphonate‐treated patients as shown in the machine‐learning model. This model is referred to in the text as the “fracture model.” Length of the horizontal bars denotes the magnitude of the linear coefficient in the machine‐learning duration model; direction and color of the bars denote coefficient sign. Red bars extending to the left denote coefficients negatively correlated with increasing likelihood of fracture; teal bars extending to the right denote coefficients positively correlated with increasing likelihood of fracture.

Nine parameters in the fracture model had estimated linear coefficients exceeding 0.2 (Fig. 6). These were included in the machine‐learning model relating bone fracture to continuous bisphosphonate treatment duration, infrared spectroscopic, and histomorphometry‐based bone‐quality parameters. The important role of bone mineralization in this fracture model is reflected by the fact that six of these nine parameters are mineral related. Parameters associated with an increasing likelihood of bone fracture were (in decreasing order of importance): increased bisphosphonate treatment duration, increased carbonate substitution for phosphate in bone mineral, increased collagen maturity, increased mineralization, decreased mineralization distribution, and increased mineral crystal length. Hip BMD and patient age were not critical parameters in the fracture model. Details regarding the estimated linear coefficients of all parameters analyzed relative to bone fractures are provided in Supplemental Fig. S2.

## 4 Discussion

New information is provided guiding use of continuous oral bisphosphonate treatment for postmenopausal osteoporosis, supporting prior work citing the usefulness of infrared spectroscopy to evaluate bone quality. This demonstrates the clinical relevance of bone mineral quality.

Relationships between bone‐quality parameters and bisphosphonate use were manifested by machine‐learning analyses only when duration was analyzed as a two‐category (1 to 8 and 9 to 14 years) variable but not as a continuous variable. This may be explained by bone quality, which changes positively with bisphosphonate use for up to 8 years of treatment, but after this period it changes negatively with continuing bisphosphonate use. This explanation is consistent with our prior finding showing increases in trabecular architecture‐based strength estimates occurring at approximately 7.5 years of bisphosphonate use, then decreases in trabecular architecture‐based strength occur with continuing use.^(^ ^8^ ^)^ It is noteworthy that these similar treatment durations, ie, 7.5 and 8 years, were discovered using different techniques quantifying different bone‐quality parameters.^(^ ^8^ ^)^ The observed 8‐year time point in the present duration model offers valuable new information identifying a treatment period after which adverse changes to bone, including atypical fractures, occur. This is consistent with observations from prior studies.^(^ ^11^, ^27^, ^28^ ^)^

Machine‐learning analyses demonstrated the importance of bone quality and underscore previous work noting the usefulness of infrared spectroscopy for assessing bone‐quality changes.^(^ ^12^, ^13^, ^29^ ^)^ Specifically, four of the seven largest linear coefficients in the duration model and six of the eight largest linear coefficients in the fracture model were bone‐quality parameters obtained from infrared spectroscopy. The relationship between bone quality and bisphosphonate treatment was observed not just for changes in mean values of some bone‐quality parameters but also in the distributions of these parameters. The latter was previously shown in a study demonstrating changes in heterogeneity of bone‐quality parameters with bisphosphonate treatment duration.^(^ ^12^ ^)^ The present study's novel application of machine‐learning methods for the holistic analysis of the large volume and variety of analyzed bone‐quality parameters confirmed prior findings and identified significant new relationships between mean values and distributions of bone‐quality parameters with bisphosphonate treatment duration.

Bone mineral purity, measured using the carbonate to phosphate ratio, was the most significant bone‐quality parameter in the bisphosphonate treatment duration machine‐learning model. This ratio quantifies the relative amount of carbonate substituted for phosphate in bone mineral crystal. Increases in this ratio with increasing bisphosphonate treatment duration may be a consequence of reduced bone turnover, resulting in older bone, longer‐lived mineral, and thus more time for carbonate to substitute for phosphate in the mineral crystal structure. The importance of the carbonate to phosphate ratio in the duration and fracture machine‐learning models is underscored by publications investigating relationships among bone quality, hormone replacement therapy, as well as proximal femoral and low‐energy fractures.^(^ ^13^, ^30^, ^31^ ^)^

The second most significant parameter in the duration model was the decrease in standard deviation of bone with low collagen maturity. Thus, with increasing bisphosphonate treatment duration, the range of values for the ratio of mature to immature collagen cross‐links decreased. As expected, the duration model showed that hip BMD was a significant parameter (third in importance) directly related to bisphosphonate treatment duration. This is consistent with increased mineralization observed in a prior study of 32 postmenopausal women with osteoporosis treated with bisphosphonates for 6.4 ± 2.0 years.^(^ ^32^ ^)^ The fourth and fifth most significant parameters of the model indicate that fewer osteoclasts and reduced activation frequency accompany longer bisphosphonate treatment durations. These findings are consistent with the known effect of bisphosphonates on bone turnover^(^ ^33^, ^34^ ^)^ and with the reported 84% reduction in remodeling activity observed in iliac crest bone samples from the previously cited study.^(^ ^32^ ^)^ The previously shown increase in mineral to matrix ratio with increasing bisphosphonate treatment duration^(^ ^12^ ^)^ was confirmed by the present study but was ranked 11th of the 15 duration‐model analyzed parameters.

The machine‐learning fracture model showed that bisphosphonate treatment duration was the most important feature predictive of the observed low‐energy fractures, ie, longer bisphosphonate treatment duration was associated with greater likelihood of fracture. This finding agrees with a study of 196,129 postmenopausal women treated with bisphosphonates showing that compared with women treated for 3 to 5 years, treatment for 8 or more years was associated with an almost fivefold increase in hazard ratio for atypical fractures.^(^ ^27^ ^)^

Relevance of bone mineral purity, quantified by the carbonate to phosphate ratio, was demonstrated by this parameter's rank (second most important) in the fracture machine‐learning model. This finding is supported by a study of 60 bisphosphonate‐naive female patients with fractures who had decreased carbonate to phosphate ratios in iliac crest cancellous bone compared with 60 sex‐, age‐, and BMD‐matched controls.^(^ ^13^ ^)^ Differences in the direction of the relationships observed in these studies may be explained by the different patient populations.

Relative bone mineralization, quantified by the mineral‐to‐matrix quality parameter, was the third most important parameter in the machine‐learning fracture model. Thus, the likelihood of low‐energy fracture in patients treated long term with bisphosphonates is directly related to increased relative bone mineralization. This finding is consistent with data showing that increased mineralization is associated with decreased fracture toughness.^(^ ^35^ ^)^

Bisphosphonate treatment is associated with increased hip BMD and consequentially reduction in fracture risk.^(^ ^36^ ^)^ Although hip BMD was the third most important parameter in the duration model and was directly related to bisphosphonate use, hip BMD was not among the top 25 parameters in the fracture model. This finding suggests that the low‐energy fractures in study patients treated with bisphosphonates for more than 8 years are those due to diminished bone quality and turnover changes occurring with long‐term bisphosphonate use.

Among the many parameters studied, patient age was not a significant parameter in the treatment duration or fracture models. Spine BMD was not included among the analyzed parameters because of the inferior fracture predictive value of spine BMD compared with hip BMD.^(^ ^37^, ^38^ ^)^

Limitations of the study are those that are characteristic of a cross‐sectional design. In addition, most patients in the study used alendronate, thus the analyses were unable to discern the effect of other oral bisphosphonates. Inclusion of bone biopsies in the study results in a study size that is small compared with those of epidemiologic studies; however, the employed sample size is sufficient to enable machine‐learning analyses to identify key bone‐quality parameters and create models that significantly relate these parameters to treatment duration and low‐energy fracture. We recognize that 22 fractures occurring in 67 patients on long‐term bisphosphonate treatment is a relatively high percentage (32.8%); however, no selection bias can be identified to explain this result.

Continuous use of oral bisphosphonates exceeding 8 years is associated with significant declines in bone mineral quality and increases in low‐energy bone fractures. These findings provide evidence for an 8‐year maximum time limit for uninterrupted treatment. Bone quality is an important aspect of skeletal health and should be considered when evaluating the efficacy of various bone metabolic therapies. Inadequate bone quality is related to increased likelihood of low‐energy fracture. Infrared spectroscopic examination of biopsied tissue samples provides critically important, but otherwise unavailable, information describing bone quality.

## Disclosures

All authors state that they have no conflicts of interest.

5

### PEER REVIEW

The peer review history for this article is available at https://publons.com/publon/10.1002/jbm4.10549.

## Supporting information


**Supplemental Fig. S1.** Graphical depiction of estimated linear coefficients of various bone‐quality relevant parameters as they relate to bisphosphonate treatment duration. This figure shows the full list of parameter features used in the machine‐learning model relating bisphosphonate treatment duration and bone quality, ie, “the duration model.” The magnitude of the estimated linear coefficient of each model parameter is proportional to horizontal length. Signs of these linear coefficients are depicted by position versus the centerline. Red bars extending to the left denote parameters negatively correlated to bisphosphonate treatment duration; blue bars extending to the right denote parameters positively correlated to bisphosphonate treatment.

**Supplemental Fig. S2.** Graphical representation showing linear coefficients of various bone‐quality relevant parameters as they relate to bone fracture in patients treated with bisphosphonates. This figure shows the full list of parameters used in the machine‐learning model relating bone fracture to bisphosphonate treatment duration and bone quality, ie, “the fracture model.” The magnitude of the linear coefficient of each model parameter is proportional to horizontal bar length. Signs of these linear coefficients are depicted by position versus the centerline. Red bars extending to the left denote parameters negatively correlated to bisphosphonate treatment duration; blue bars extending to the right denote parameters positively correlated to bisphosphonate treatment duration.
