# Supplementary figures and images for: Bone Quality and Fractures in Women With Osteoporosis Treated With Bisphosphonates for 1 to 14 Years
Source: JBMR Plus. 2021 Sep 21;5(11):e10549. doi: 10.1002/jbm4.10549 (PMC8567493; doi:10.1002/jbm4.10549)

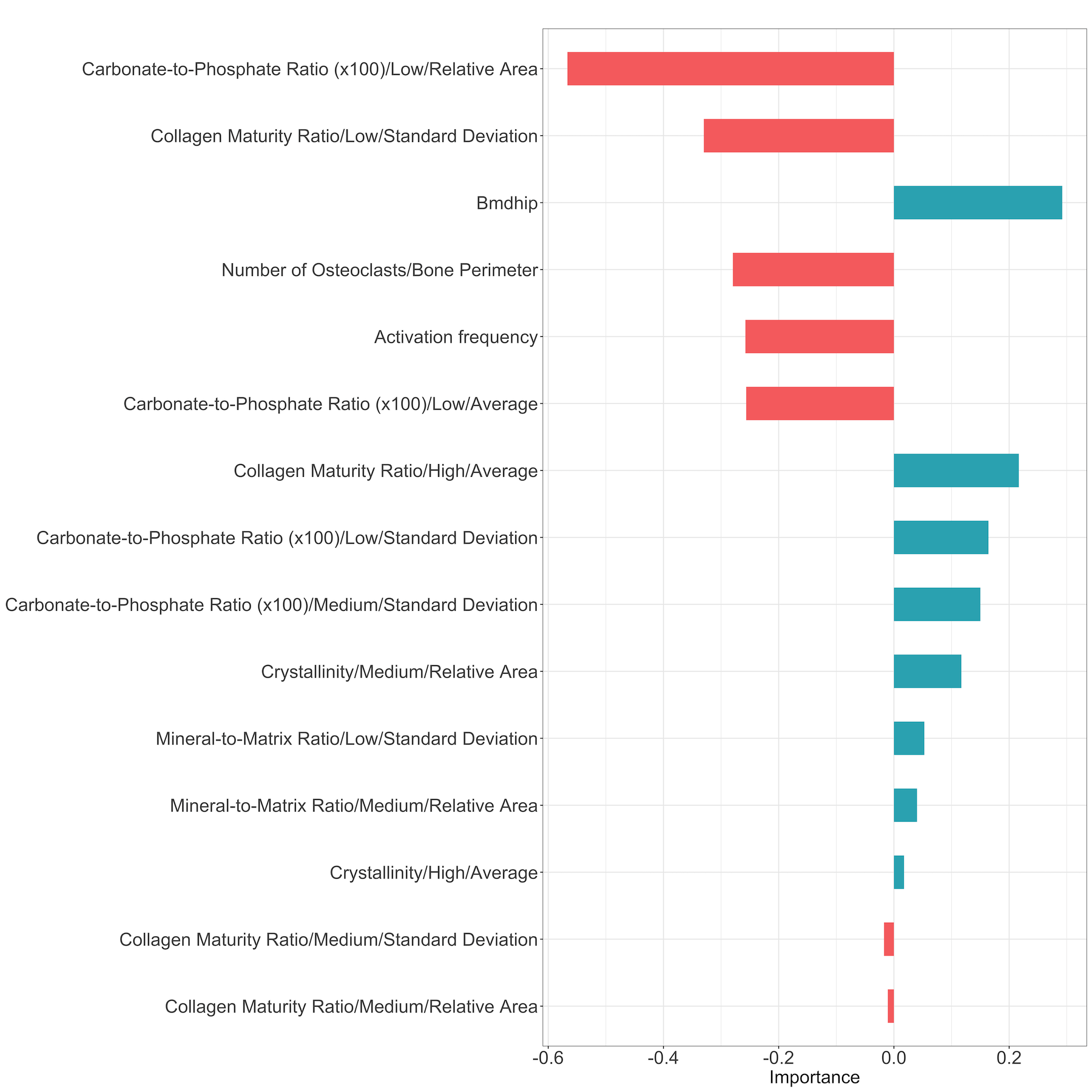

Supplement: Supplementary file 1 — Supplemental Fig. S1. Graphical depiction of estimated linear coefficients of various bone‐quality relevant parameters as they relate to bisphosphonate treatment duration. This figure shows the full list of parameter features used in the machine‐learning model relating bisphosphonate treatment duration and bone quality, ie, “the duration model.” The magnitude of the estimated linear coefficient of each model parameter is proportional to horizontal length. Signs of these linear coefficients are depicted by position versus the centerline. Red bars extending to the left denote parameters negatively correlated to bisphosphonate treatment duration; blue bars extending to the right denote parameters positively correlated to bisphosphonate treatment. [file JBM4-5-e10549-s001.tiff]

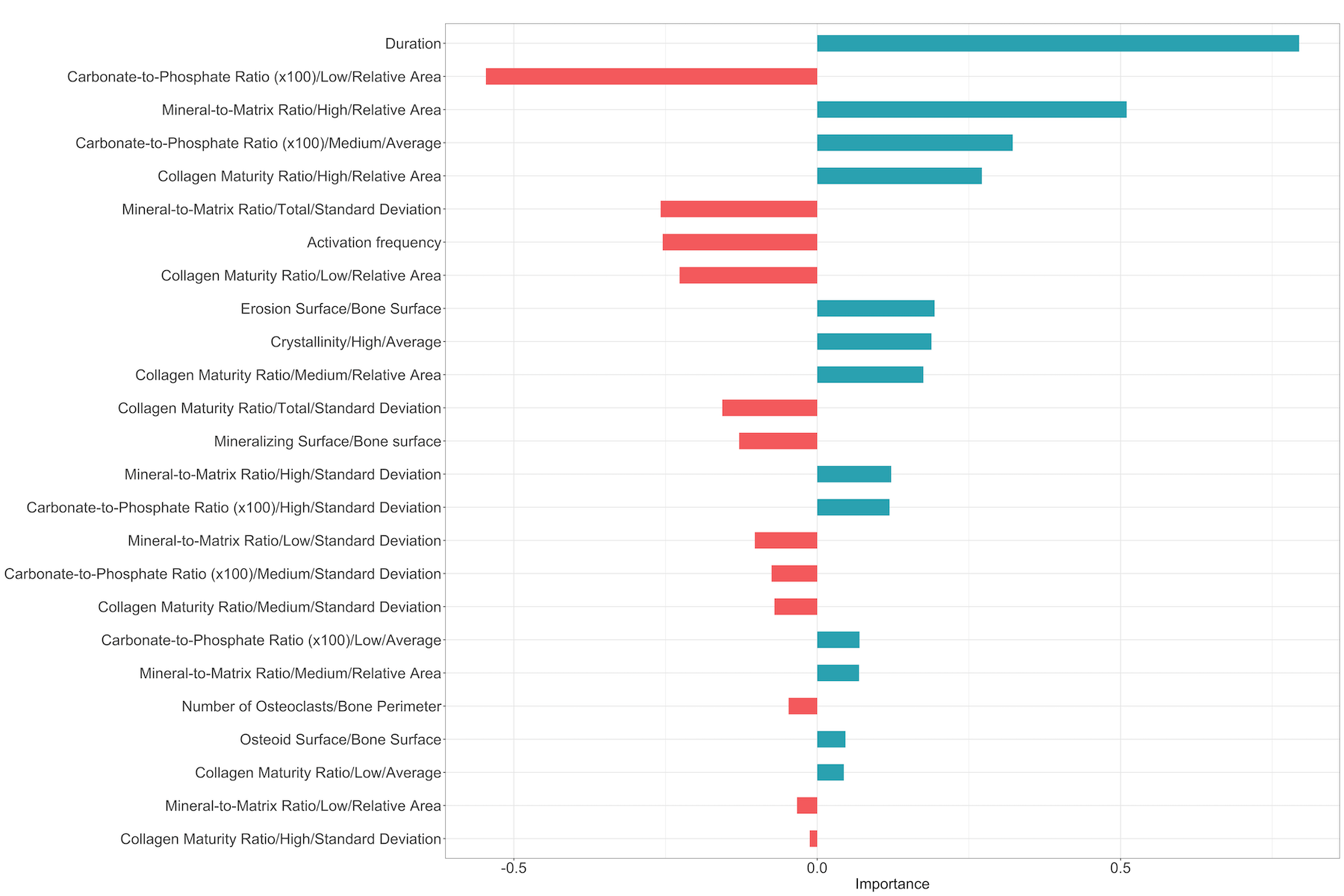

Supplement: Supplementary file 2 — Supplemental Fig. S2. Graphical representation showing linear coefficients of various bone‐quality relevant parameters as they relate to bone fracture in patients treated with bisphosphonates. This figure shows the full list of parameters used in the machine‐learning model relating bone fracture to bisphosphonate treatment duration and bone quality, ie, “the fracture model.” The magnitude of the linear coefficient of each model parameter is proportional to horizontal bar length. Signs of these linear coefficients are depicted by position versus the centerline. Red bars extending to the left denote parameters negatively correlated to bisphosphonate treatment duration; blue bars extending to the right denote parameters positively correlated to bisphosphonate treatment duration. [file JBM4-5-e10549-s002.tiff]
